# Mysterious long-living ultrahigh-pressure or secondary impact crisis

**DOI:** 10.1038/s41598-020-59520-3

**Published:** 2020-02-13

**Authors:** T. G. Shumilova, A. A. Zubov, S. I. Isaenko, I. A. Karateev, A. L. Vasiliev

**Affiliations:** 1 Institute of Geology, Federal Research Centre “Komi Science Centre of the Ural Branch of the Russian Academy of Sciences”, Syktyvkar, Russia; 2 National Research Center “Kurchatov Institute”, Moscow, Russia; 3 Moscow Institute of Physics and Technology (State University), MIPT, Dolgoprudny, Moscow Region Russia

**Keywords:** Environmental impact, Mineralogy, Mineralogy, Core processes, Structure of solids and liquids

## Abstract

High-pressure glass has attracted interest in terms of both its fundamental state under extreme conditions and its possible applications as an advanced material. In this context, natural impact glasses are of considerable interest because they are formed under ultrahigh-pressure and high-temperature (UHPHT) conditions in larger volumes than laboratory fabrication can produce. Studying the UHPHT glasses of the unique giant Kara astrobleme (Russia), we found that the specific geological position of the UHPHT melt glass veins points to an origin from a secondary ultrahigh-pressure (UHP) melt according to the characteristics of the host suevites, which suggest later bottom flow. Here, we propose a fundamentally novel model involving an upward-injected UHP melt complex with complicated multi-level and multi-process differentiation based on observations of the UHP silica glass, single-crystal coesite and related UHP smectite that crystallized from an impact-generated hydrous melt. This model proposes a secondary UHP crisis during the modification stage of the Kara crater formation. The results are very important for addressing fundamental problems in fields as diverse as condensed matter states under extreme pressure and temperature (PT) conditions, material and geological reconstructions of impact structures, water conditions in mineral substances under UHP conditions in the deep Earth, and the duration and magnitude of the catastrophic effects of large asteroid impacts.

## Introduction

The structure and properties of disordered substances have been studied for a long time and used in a wide range of applications. The behaviour of such substances under extreme conditions, including intense compression, is particularly interesting and is actively studied both theoretically and experimentally^1–7^. High-pressure glasses have drawn interest both fundamentally as a material formed under extreme conditions and as a novel compound that could be used in new applications^8,9^.

The behaviour of disordered and weakly ordered systems under compression has been studied in microvolumes via diamond anvil. Typically, the compression process is performed at pressures of up to 100 GPa and sometimes higher. Furthermore, the experiments are typically performed at room temperature^1,4–6,9^. Only a few experiments combining both high pressures and high temperatures have been carried out. However, such studies are intriguing because the simultaneous effects of temperature and pressure can produce profound internal changes in a substance. For example, SiO_2_ glass has a significantly higher density and strength at a pressure of approximately 8 GPa and a temperature of 1100 °C than under cold compression^10^. The most important properties expected from these glasses are the heat capacity, hardness, unusual optical properties and other physical properties, which can be used in high-tech materials and technologies, including high-energy lasers, microelectronics and innovative optics. Glasses with microstructural features at a nanoscale are potential basic matrices for such materials^11–13^.

### Natural impact glasses

In this context, natural impact glasses are very interesting. Depending on the specific impact event, the compression level in large astroblemes, determined by the size of the falling body, is typically greater than 10 GPa. Objects in which impact glass has formed undergo pressures of 35–90 GPa and accompanying temperatures of up to 3000 °C. Moreover, the pressures in the area of the greatest impact influence can reach one hundred gigapascals at temperatures of up to 5000 °C^14–16^.

These conditions experienced by natural impact objects cannot be achieved experimentally in bulk matter, and at present the artificial production of such material is difficult for material applications or fundamental studies. Therefore, natural impact glasses are a valuable material for studies and have many potential applications. However, until now, impact glasses have been neglected and considered mainly as indicators of impact events^15–21^. The microstructures of natural high-pressure glasses have only been slightly discussed^11,22–24^, and the comprehensive study of the structure and properties of natural high-pressure glasses remains practically unexplored.

Among the numerous known impact structures, the Kara astrobleme (Russia) is one of the largest astroblemes in which ultrahigh-pressure and high-temperature (UHPHT) glasses have been found^25^. Based on the SiO_2_ phase state diagram^15,19^, previous experiments, the definition of an emerging shock temperature during the impact, and theoretical calculations^26^, the observed coesite in the SiO_2_ glasses of the Kara astrobleme^20,25,27^ correspond to an impact-induced melt temperature greater than 1700 °C. Thus, the impact melt products of the Kara astrobleme are very attractive from not only a fundamental point of view but also considering the probable practical use of impact glass^25^.

During field work in the Kara astrobleme in 2015 and 2017 by the Russian team of the Laboratory of Diamond Mineralogy (IG FRC KomiSc UB RAS, Russia), fundamental geological features of the melt impactites that have not been described in any other impact sites or modelling efforts were observed for the first time in this impact site^14,28–32^. In this paper, for the first time, we describe an upward-injected impact melt complex containing UHPHT impact glass veins and vein-like bodies with melt-crystallized coesite and impact diamonds cutting the hosting suevites. These observations will improve the development of models of large astrobleme formation. A fundamentally novel model of the UHPHT melt impactites is presented based on the repeated UHP process resulting from the back-stress reaction of the target.

### Kara astrobleme geological features

The Kara astrobleme is a crater with a diameter of 65 km coupled with a smaller impact astrobleme, Ust’-Kara, with a diameter of 25 km. The impact craters are positioned near each other in the northeast of the European part of Russia and belong to the Pay-Khoy Ridge structure, Yugor Peninsula, Russia (Supplementary Fig. 1). The Kara astrobleme is adjacent to the Kara Sea, while the Ust‘-Kara crater is generally underwater. At present, the Kara astrobleme presents a shallow depression in the landscape but is well recognized with gravity and magnetic fields^33,34^.

Before the 1970s, the accepted origin of the Kara depression has volcanic origin. The impact origin was considered for the first time in 1970 by P.S. Voronov, who shared his idea with the public media (press). The hypothesis was proven by V.L. Masaitis in 1971 and was shortly thereafter accepted and further developed by industrial geologists headed by M.A. Maslov and G.Ya. Ponomarev^33^; subsequent scientific studies have been carried out since the 1980s^18,21,33,35–37^. Most recently, the full complex of silica polymorphs, including high-pressure coesite after diapectic quartz and low-pressure postimpact phases, in the Kara impactites has been described^20^. The structure of the Kara impact crater, as recognized by geophysical data, corresponds well to the fundamental features of large astroblemes with a central uplift^16,33^. According to the most recent deep geophysical observations, the Kara depression is emplaced entirely in crustal lithologies and does not have any mantle roots^38^. Thus, the nature of the impact of the Kara depression has been unambiguously proven. According to ^40^Ar–^39^Ar on the basis of melt rock measurements, the giant Kara impact event occurred at 70.3 ± 2.2 Ma^36^.

In 1976, the Ust’-Kara astrobleme was identified as an independent impact crater with an origin age similar to that of the Kara astrobleme^33^. At present, it is accepted that the Kara and Ust’-Kara astroblemes resulted from the same impact event produced by a single bolide that broke up just before contact with the Earth’s surface.

**Figure 1:**
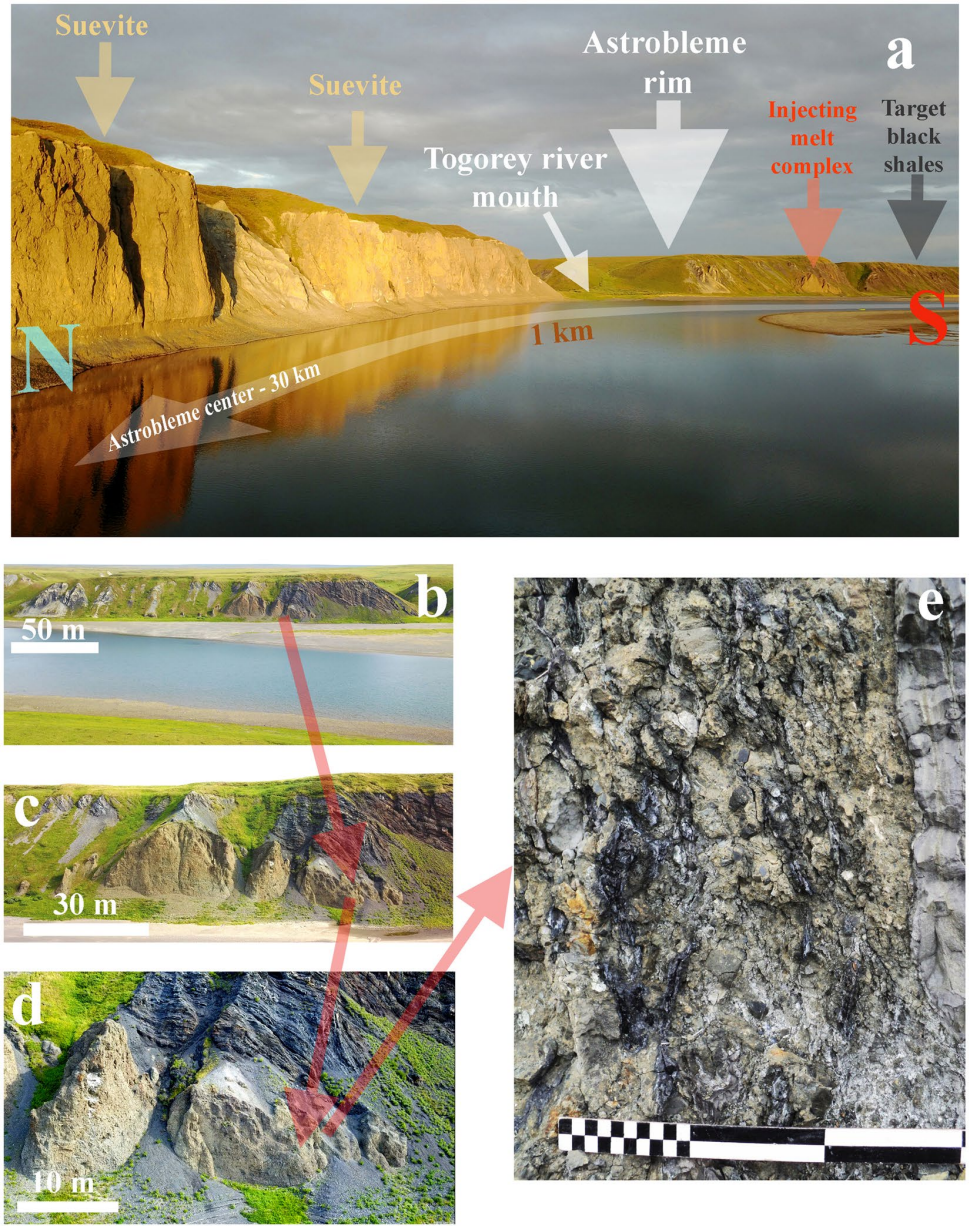
Photographs of outcrops of crater suevites and the upward-injected UHPHT melt complex in the Kara astrobleme: (**a**) – overview of the rim of the astrobleme, right bank of the Kara River, (**b**) – upward-injected UHPHT melt complex; (**c**) – upward-injected UHPHT melt massif; (**d**) – edge of the upward-injected UHPHT melt massif with glass veins; (**e**) – UHPHT melt glass veins, the smallest scale bar corresponds to 1 cm; the images in (**a–d**) were collected by drone.

The geological composition was described in detail by M. S. Machshak^33^ and M. A. Shishkin *et al*.^34^, who noted that the Kara region belongs to the Pay-Khoy subzone of the Zilairo-Lemvinskaya zone, with bathyal sediments corresponding to the Sylovayachinskaya (D_3_–C_1_), Karskaya (C_1_) and Karasilovaya (C_2_–P_1_) units. According to M. S. Machshak^33^ and M. A. Shishkin *et al*.^34^, the target in the Kara astrobleme region presents two structural levels: late Proterozoic sediments (lower level) and Paleozoic sediments (upper level) (Fig. 1). The upper structural level contains a 3.5 km thick package of Ordovician-Carboniferous and early Permian sediments. Many layers of sediments are enriched in carbonaceous matter with different levels of coalification, providing as a source of carbon for the diamond formation in the Kara astrobleme^27,37^.

In the Kara astrobleme, several types of impactites have been previously discovered, including allogenic lithic breccias, massive melt rocks and suevites, as described elsewhere^33,34,39^. Here, we focus on the description of the discovered upward-injected UHPHT impact melt complex.

### Upward-injected melt complex

On the basis of our field observations in 2015 and 2017 followed by detailed laboratory studies of the solidified melt material, we have identified an *intrusive melt complex* composed of different portions of an *ascending impact melt intruding breccia and suevites* for the first time in the Kara astrobleme*.* The complex has been discovered at the edge of the impact crater at the modern erosion level. We describe the complete profile of the discovered intrusive complex on both banks of the Kara River at the present rim of the astrobleme (Fig. 1). The geological observations have been provided via outcrop descriptions and sampling and remotely acquired observations via drone-based videographic and large-scale photographic observations. Here, we present the discovered complex for the first time.

We start the description with the melt vein system that intruded into the suevite massif because understanding this feature helps with the analysis of the upward-injected melt complex overall.

#### Intrusive melt veins

On the right bank of the Kara River, at the rim of the Kara astrobleme, we found a suevite massif consisting of a high content of sandstone lithic clasts and melt clasts. The geometry of the suevite massifs and their spatial relations to the target sedimentary rocks were analysed with 3D observations under field conditions using a drone (Fig. 1c). According to our manual field studies and drone-based “bird’s-eye view” videographic and photographic observations, we conclude that the suevite massif is overlain by black shales of the sedimentary target, which are upturned, forming an uplifted edge due to the crater’s radial excavation (Fig. 1c,d), as has been described at the crater rim in numerous impact crater models^29^.

**Figure 2:**
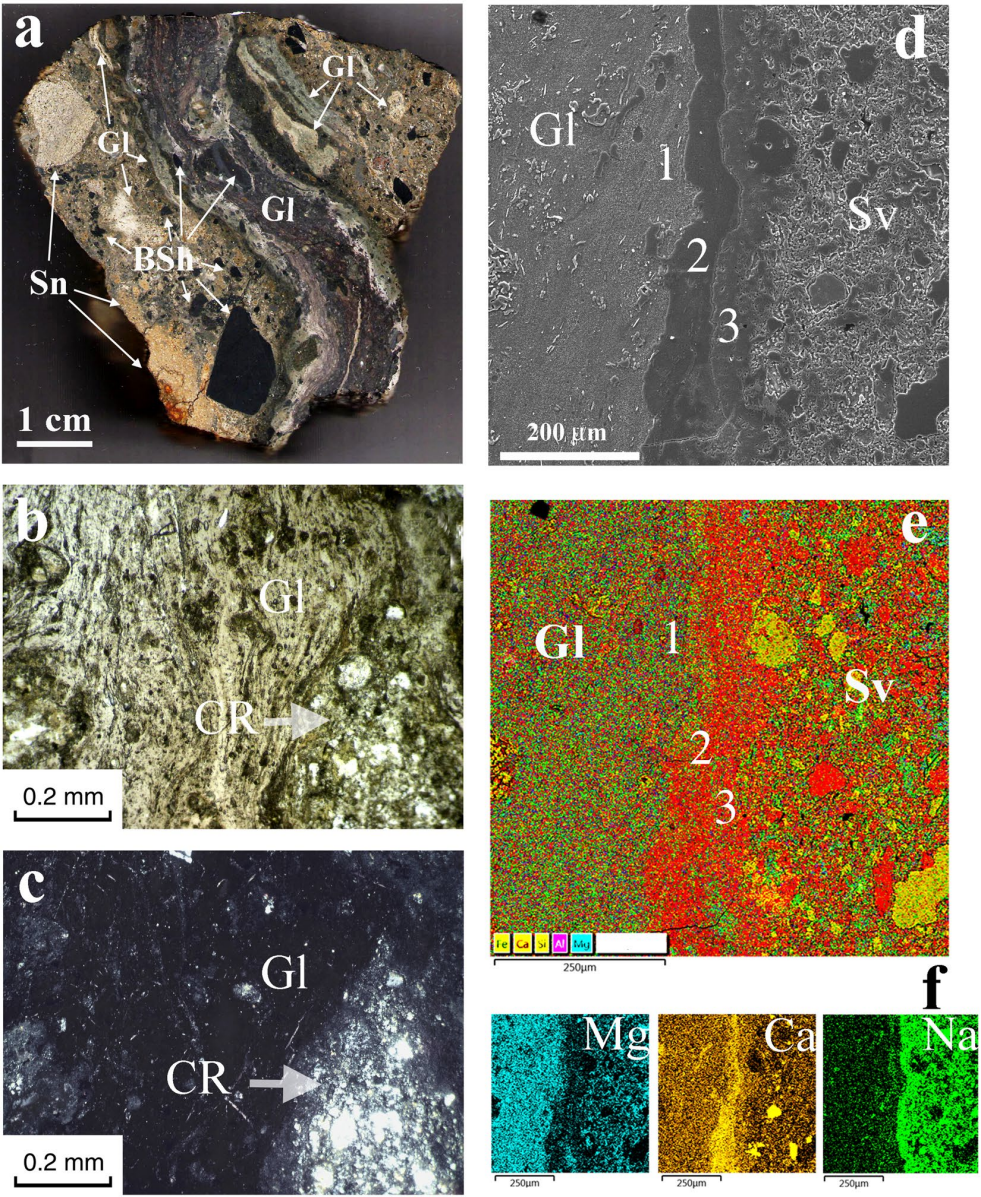
Composition of the UHPHT melt glasses: (**a**) – cross-cutting vein relative to the host suevite (polished plate, optical image); (**b,c**) – optical images of the central part of the melt vein, transmitted light with parallel polarizers (**b**) and with crossed polarizers (**c,d**) – secondary electron mode of scanning electron microscopy (SEM) image of the quenching zone between the UHPHT melt and the host suevite; (**e**) – microprobe multi-element map of the quenching zone; (**f**) –elemental maps showing strong variation in element concentrations in the quenching zone. Gl - glass, Sv - suevite, BSh - black shale fragments, Sn - sandstone fragments, CR - rock clast; 1, 2, 3 - subzones of the quenching zone.

The general feature of the described suevite massif is the presence of the cross-cutting, tight stockwork-like system of impact melt glass in thin upward-injected veins of lilac, grey and (rare) black colours (Figs. 1e, 2a). The glass veins are 1–10 cm thick and twist, with visible parts up to 1.5 metres in length on the outcrop surface. Using the drone video, we observed the impact glass veins from the bottom up to the top of the suevite massif: the system of subparallel thin bodies can be accurately identified by their colour in contrast to the beige host suevite (Fig. 1e). The glass veins have white endo- and exocontacts in quenched zones of the impact melt as the heating caused partial melting of the host suevite. This pattern is clearly visible in microprobe studies and parallel scanning electron microscopy observations (Fig. 2).

A very specific feature of the found upward-injected melt glass veins is the presence of ultrahigh-pressure (UHP) phases, such as impact diamonds and melt-crystallized coesite^25,27,39^. The latter suggests that the cross-cutting melt veins could not have been associated with any hydrothermal activity and must have a high-pressure melt origin, which we discuss below.

**Figure 3:**
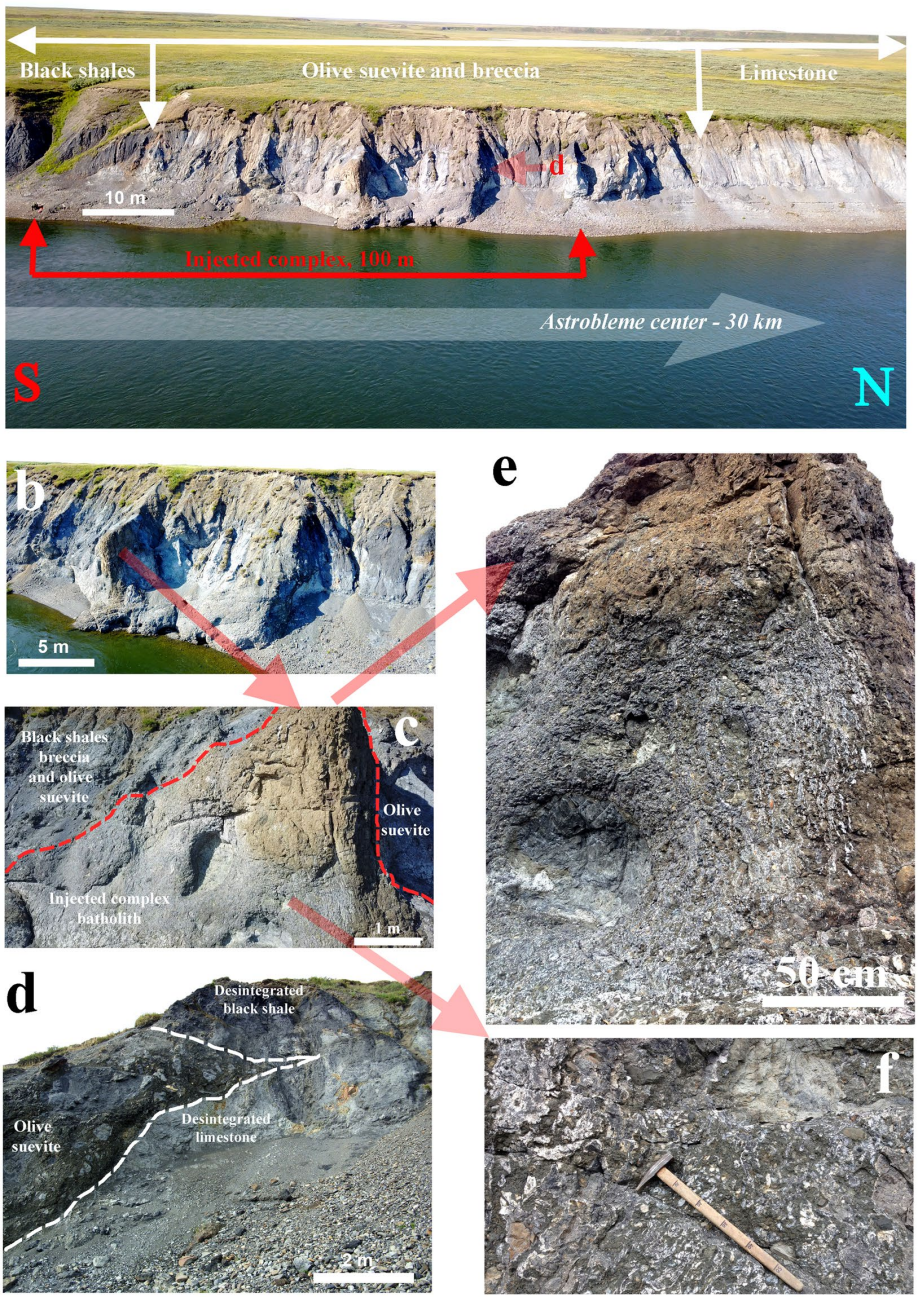
Images of outcrops of the upward-injected UHPHT melt complex in the Kara astrobleme: (**a**) – an overview of the rim part of the astrobleme, left bank of the Kara River, **(b**) – the upward-injected UHPHT melt complex with a central melt body; (**c**) – location of the central melt body relative to the olive-coloured suevite; (**d**) – the injection of wedge-shaped suevites (olive-coloured suevites) into a weak zone between limestones (bottom layer) and black shales (upper layer); (**e)** – the vein-like texture of the differentiated upward-injected melt body; **(f**) – mottled texture of the partially differentiated UHPHT impact melt; (**a–c**) – images collected by drone.

The *intrusive impact melt body* was discovered on the left bank of the Kara River together with intrusive thin melt glass veins at the southern edge (left side) of the outcrop and bulk melt dykes at the northern edge (right side) of the outcrop (Fig. 3). The melt body is up to 100 m long and up to 8 m high in the highest part of the observed outcrop. The specific feature of the melt body is originally partly liquid melt forming an unusual vein-like texture due to the movement of immiscible melt components (Fig. 3e,f). The melt body varies in colour depending on the initial melt differentiation. The undifferentiated parts are brown, whereas the differentiated parts have a mottled characteristic (Fig. 3f) and are predominantly lilac in the central zones and almost white at the outer parts of the glass vein-like bodies. The latter can be observed as a subparallel system of thin glass vein-like bodies or as irregular and large-area shapes depending on their orientation and were probably controlled by the melt flow velocity – the faster the melt flow velocity was, the straighter the glass vein-like bodies (Fig. 3e,f). The bulk melt body is characterized by a flow texture, a high crystallinity and a high porosity. At the same time, the accompanying melt veins at the very edge of the intrusive upward-injected complex on the left bank of the Kara River have a pore-free texture and a low crystallinity. The latter have UHPHT features, such as melt-crystallized single-crystal coesite and after-coal diamonds.

The massive melt body includes some large blocks of the sedimentary target limestone with a rounded shape resulting from impact-induced melting. The thin veins contain rare small fragments of other sedimentary clasts, including clasts of sandstones and black shales. The surfaces of the fragments partially melted, and the small clast fragments are thermally altered throughout.

### UHPHT glasses

**Figure 4:**
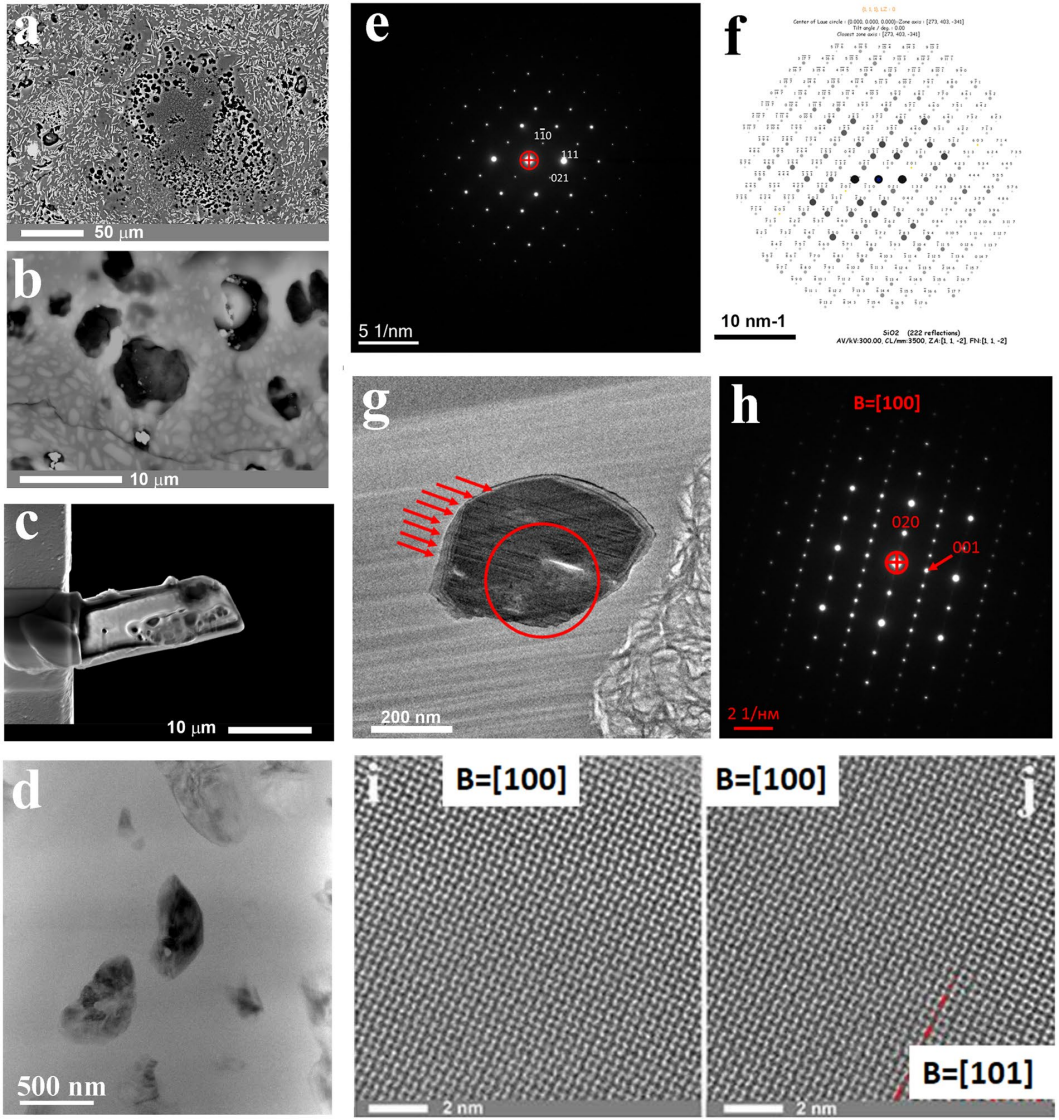
Single-crystalline coesite within a UHPHT impact glass vein: (**a–c**) – SEM images, (**a**) –coesite-bearing liquated SiO_2_ glass drop within partially crystalline aluminosilicate glass, the light grey microcrystals are augite; (**b**) –coesite crystals (light grey) and smectite within silica glass; (**c**) –lamellae prepared by focus ion beam milling of the coesite crystals and smectite; (**d**) –coesite crystals within silica glass, bright field transmission electron microscopy (TEM) image; (**e–j**) –coesite analysis: selected area electron diffraction pattern (**e**) (the corresponding d-spacings are presented in Supplementary Table 1) and its simulated pattern (**f**), a magnified view of (**f**) with well-resolved indexes is presented in Supplementary Fig. 2; the microstructure of the coesite with twins (**g**) and its selected electron diffraction pattern (**h**); high-angle annular dark field (HAADF) high-resolution scanning transmission electron microscopy (HR STEM) images of a perfectly crystalline part of a coesite particle, shown in g (**i**) and a twin boundary (**j**).

**Figure 5:**
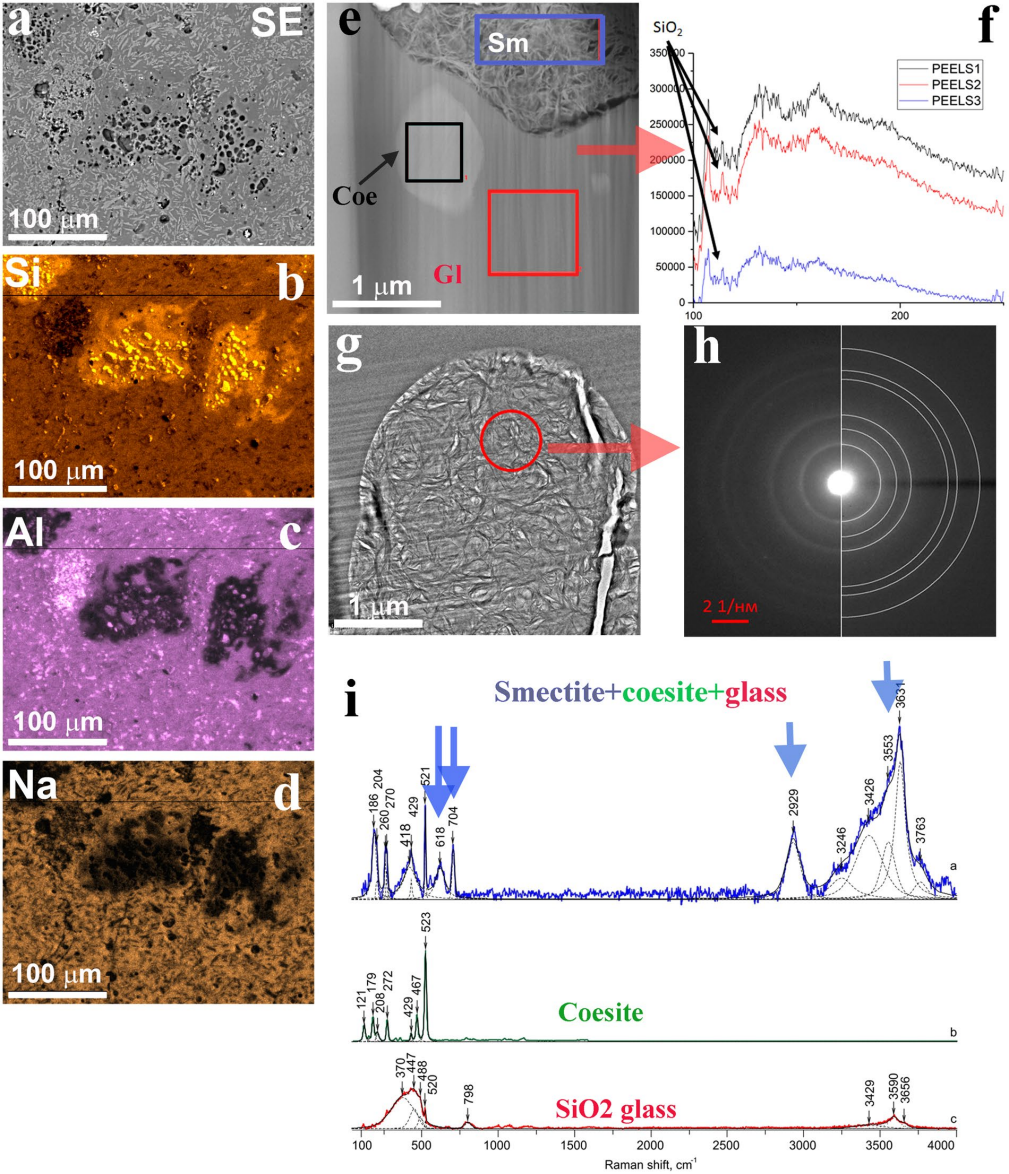
Smectite-coesite-UHPHT glass association: (**a**) – secondary electron (SE) SEM image of the UHPHT glass silica drops within the host aluminosilicate glass, (**b–d**) – microprobe mapping data of the region **(a**) by element – Si (**b**), Al (**c**), Na (**d**); (**e)** – smectite aggregate (Sm) and coesite crystal (Coe) within the UHPHT silica glass (Gl), bright field TEM image; (**g**) – smectite spherical aggregate within the UHPHT silica glass, bright field TEM image; (**f)** – parallel electron energy loss spectrometry (PEELS) spectra from the regions in (**e**), red – the UHPHT silica glass, black – coesite; blue – smectite; (**h)** – electron diffraction pattern of smectite within the host glass of the selected rounded area (**g**); (**i**) – measured Raman spectra of smectite-coesite-SiO_2_ growth, pure coesite and the host SiO_2_ glass.

The glass within the injected melt complex is mostly aluminosilicate (feldspar) with widely spaced 2–5 µm augite microcrystals in varying proportions in the amorphous glass matrix (Fig. 4). The aluminosilicate glass is accompanied by numerous liquated silica drops with single-crystal coesite and water-rich aluminosilicate melt drops crystallized to smectite (Fig. 5), similar to clay crystallization from impact-generated hydrous melt^40^. Rare impact carbon particles are present throughout both the aluminosilicate and silica glasses.

Based on our observations^27^, the initial UHPHT melt differentiated via a complicated sequence of processes during the post-impact decrease in pressure and temperature, including several stages of liquation, crystallization and glass condensation.

High-resolution transmission electron microscopy (HRTEM) images and Raman spectra indicate that the UHPHT silica glass is amorphous without recognizable short-range order (Fig. 5). The total chemical composition of the aluminosilicate and phase state based on X-ray diffraction are presented in our previous work^25^. In the same paper, it was noted that the glass veins had unusual 8-fold co-ordination sites of Fe^2+^, which had not been described before in either synthetic or natural glasses. The co-ordination site feature was assumed to be a special feature of the high-pressure history of the UHPHT glass structural elements.

### UHP crystalline phases

*After-coal diamonds* were enriched from the vein UHPHT glasses by thermo-chemical dissolution of the host glasses via enrichment technology^39^. The diamonds present two varieties: more common brown paramorphs after organic relics and less common white sugar-like grains. Numerous carbon particles were found directly within polished sections of the UHPHT glasses, either *in situ* in a general glass matrix or within liquated silica drops accompanied by coesite (Fig. 4). The diamonds are similar in shape to those described in^39^ but exhibit less distinct morphologies, most likely due to the UHPHT melt conditions before its solidification to glass.

*Coesite* within the described UHPHT glasses was present in the form of single-crystalline particles within only differentiated pure silica or in a few Al_2_O_3_ drops surrounded by the general aluminosilicate mass of the glass veins. The coesite particles exhibited a rounded shape, probably due to the effects of the hot UHPHT silica melt treatment. The parameters of the crystal lattice unambiguously match those of coesite (Fig. 4; Supplementary Table 1; Supplementary Fig. 2). High-angle annular dark field (HAADF) scanning transmission electron microscopy (STEM) data demonstrated that a coesite particle mostly exhibited a single-crystalline structure (free of mechanical defects) with rare polysynthetic twins intersecting the whole coesite grain (Fig. 4g–j). The latter points to the growth nature of the twins^41^. Within the liquated silica drops, the coesite exists as numerous particles that vary in size.

#### Smectite (crystallized hydrous aluminosilicate melt)

Numerous smectite occurrences within the UHPHT pure SiO_2_ glass were found (Fig. 5a). These smectite aggregates adopted a spherical morphology that is clearly visible in transmission electron microscopy (TEM) images (Fig. 5a,b). These aggregates were up to 10 µm in size. The particles consist of flake-like aggregates (Fig. 5b) and exhibit the typical morphology of illite-smectite layered intergrowths (Fig. 5c) at higher magnification^42^. HRTEM images (Fig. 5d,e) demonstrate that the distances between the lattice fringes in these flakes vary in the range of 0.8–1.3 nm (see Fig. 5e), which is also observed in illite-smectite crystals. The HRTEM image (Fig. 5f) and the corresponding Fourier power spectrum (Fig. 5g) indicate the presence of illite^43^, as observed in the [110] zone axis. The simulated electron diffraction pattern (Fig. 5h) is similar to the Fourier power spectrum. The energy-dispersive X-ray spectroscopy (EDX) data (Fig. 5i) demonstrate spectra that are characteristic of the chemical contents of mixed smectite. We found relatively high Fe contents (up to 3 at.%), and similar values have been observed previously in volcanic glass^44^. Following the observed morphological relations between the host silica drops and the smaller drops of smectite (Fig. 5a, Supplementary Fig. 3 and Table 2), we conclude that the latter is a result of the *condensation of liquated hydrous melt* within silica melt/glass. The phase was studied in polished thin sections by Raman spectroscopy via a combination of HRTEM, electron diffraction, EDX and electron energy loss spectroscopy (EELS) in focused ion beam foils (Fig. 5a; Supplementary Fig. 3 and Table 2).

Within the UHPHT glass, clay usually exhibits a high degree of luminescence, and the measured Raman spectrum corresponds well to montmorillonite (Fig. 5i). Although the possibility of clay formation directly from impact-generated hydrous melt has been described by G.R. Osinski^40^, the numerous tight spatial intergrowths of smectite with coesite within silica glass are observed here for the first time. The unusual UHPHT-related clay can help elucidate the role of water in the formation of hydrous impact glass.

To understand the role of water^45^, it is necessary to consider that a high water content in the sedimentary target and in the initial impact melt could further increase the pressure^16^ and decrease the viscosity, possibly resulting in a melt with a high injectability.

Currently, the PT conditions of smectite crystallization are not clear for impact-generated hydrous melts because there are no observed specific relationships with mineral phases apart from the pure SiO_2_ host glass. The high water content within the impact-generated hydrous melt could have provided the necessary conditions for further solidification and crystallization, such as an increased pressure and reduced temperature.

### Mysterious UHP

#### New findings in astrobleme structure

Considerable data on large impact crater formation have been published^14,21,28–31,33,46–51^. According to the numerous models, the UHP shock stage at impact has a very short duration, ranging from less than a second to generally no more than 10 seconds, according to numerous experimental works^48^. Based on our observation data from the Kara astrobleme, we discuss new potential astrobleme structural models and the duration of UHP conditions induced by the impact process.

First, we would like to draw attention to an unusual finding demonstrating that at the Kara astrobleme rim, olive-coloured suevite was injected between layers of the target black shales and limestone (Figs. 1, 3). This finding agrees with Th. Kenkmann’s astrobleme structural model involving pre-existing weaknesses between target layers in the rim (2014) and the subsequent numerical model^48^. However, in our case, the principal novelty is the possibility of suevite injection instead of simply material^29,48^. The next unusual fact is connected to the observed cross-cutting subvertical position of the UHPHT glass melt veins in relation to the host olive-coloured suevite injected between the target layers (Figs. 1–3). A quenching effect between the glass and the host suevite is observed at all levels of analysis, from field observations to microscopic studies (Figs. 1, 2). The analysis details allow identification of outer and inner quenching zones at the boundaries of the glass veins (Fig. 2). The cross-cutting position and the quenching effect of the UHPHT glass veins point to upward-migrating melt movement and solidification after the host olive-coloured suevite.

#### UHP nature of the glass veins

The other important features of the glass within the veins and vein-like bodies are the presence of single-crystalline coesite and its growth twins crystallized from silica melt, pointing to the UHPHT nature of this mineral. Here, it is necessary to mention the observed liquation within the UHP melt to silica/aluminosilicate immiscible liquids, as this feature is indicative of high-temperature conditions^27^. To avoid the metastable coesite recrystallizing into stable SiO_2_ polymorphs, the very hot silica melt had to be in a UHP state.

#### Repeated UHP conditions

All the facts described above demonstrate a need for a much longer time for the partial melt differentiation through liquation and diffuse crystallization than usually accepted for the duration of the UHP stage during a direct impact event. As mentioned above, a typical impact UHP stage lasts just a fraction of a second. The common melt rocks in the Kara astrobleme, including massive melt bodies and clastic glasses^25^, do not feature evidence of long-duration UHP conditions, do not contain melt-crystallized coesite and have different chemical compositions. Therefore, the initial impact melt did not experience long-duration UHP conditions and cannot have been the source of the studied UHPHT glass veins. Thus, the reason why the UHP conditions lasted longer than the ultrafast shock pressure effect is still a mystery. Based on our observations at the Kara astrobleme, described above, we propose a model involving probable repeated generation of UHP conditions.

**Figure 6:**
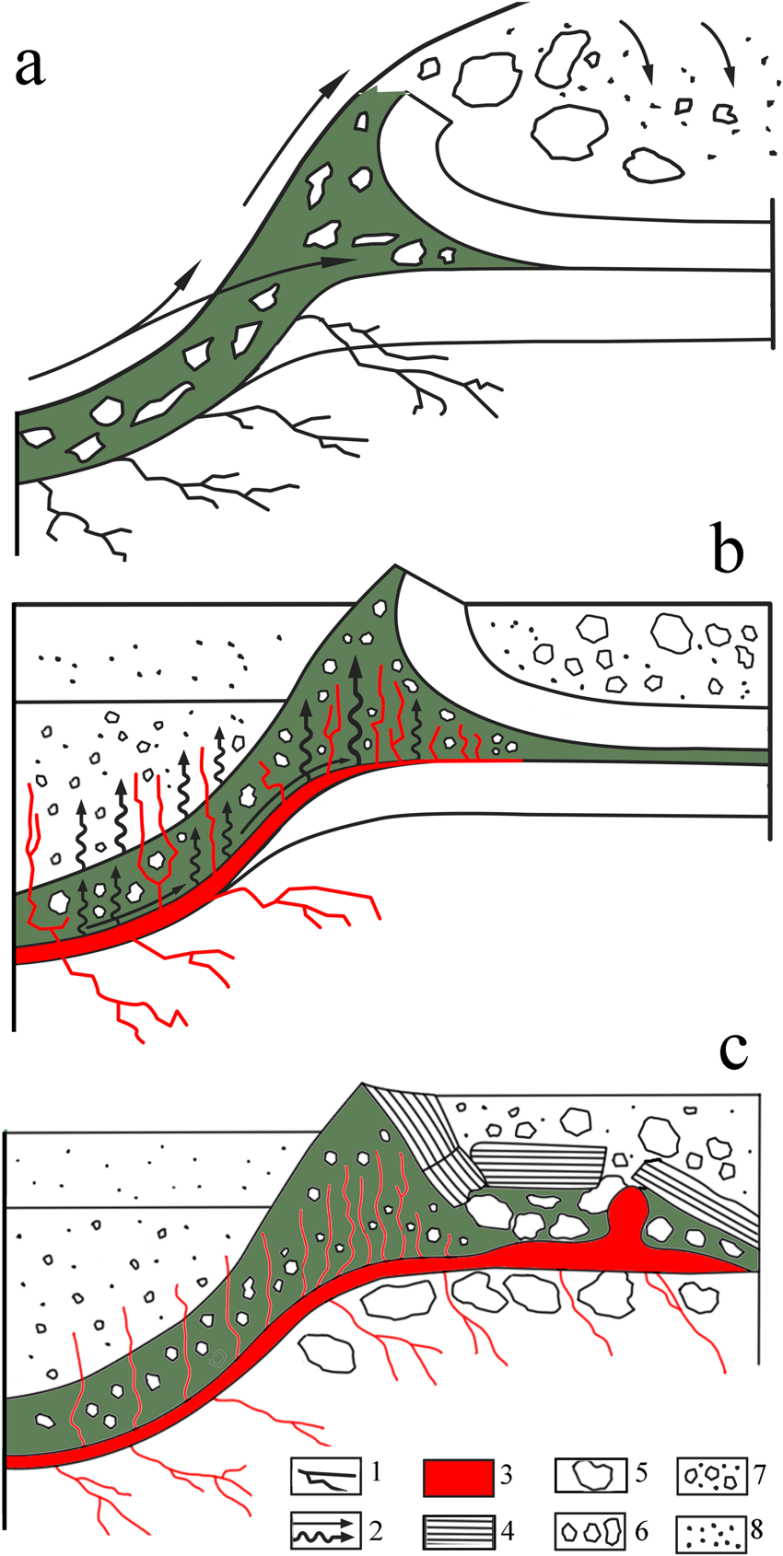
Schematic model of the formation of the injected UHPHT complex in the rim of the Kara astrobleme: (**a**) – principal trajectories of the bottom flow and outer fallout with injection of bottom-flowing wedge-shaped suevites (olive-coloured suevites) into weak zones between limestones (bottom layer) and black shales (upper layer); (**b**) – the UHPHT melt moving radially during the uplift produced by the back-stress target reaction in the intermediate stage of transient crater collapse; (**c**) – final stage of the injected UHPHT complex formation with melt veins and a batholith (bulk melt body). Legend: 1 – cracks; 2 – directions of ejecta; 3 – impact melt with blocks of disintegrated sedimentary target in ejecta, breccia and suevites; 4 – gigantic blocks of black shales; 5 – large blocks; 6 – medium blocks; 7 – small blocks; 8 – dust.

It has been proposed that large impacts can be followed by a later reaction of the target^16,52^, which has been called a secondary crisis^47,53–57^. A secondary crisis may not explain local mantle volcanism^46^ but may explain subsurface crustal magmatism^46,47^ and central uplift formation^16,52^. The observed geological features of the UHPHT glass veins could be the result of aftershock processes, such as an elastic target response followed by melt flowing along the crater during the intermediate stage of transient crater collapse. Thus, the melt may have been injected into cracks, between breccia blocks, through disintegrated sedimentary layers, and along sedimentary contacts^29,48^, as observed in the rim of the Kara crater (Figs. 1, 3). Based on our field observations and detailed laboratory-based studies, we propose a model for the upward-injected UHP intrusive complex (Fig. 6), in which the UHPHT melt intruding the suevite resulted probably from a back-stress reaction during the transient crater formation stage and uplift of the target strata. The duration of the post-shock high-pressure stage is not quite clear, but according to our observed data, it must have been longer than one second but probably not as long as previously posited^47^. Thus, this duration will be the focus of future studies.

## Conclusion

In summary, we propose that the obtained data on the recently discovered UHPHT impact glass veins in the Kara astrobleme demonstrate the formation of an upward-injected melt complex. The studied UHPHT glass veins and vein-like bodies suggest a complicated formation of the unusual complex comprising amorphous UHPHT silica glass with related smectite crystallized from the impact-generated hydrous melt, diamonds and single-crystalline coesite. The presented data point to the possibility of a relatively long duration of UHP conditions for impact melts in giant astroblemes. This subject will be considered in the future development of impact models, geological reconstructions of giant impact structures and estimates of the conditions of impact structure formation, aiming to understand the environmental implications, including the possibly of multiple phases of UHP conditions. It is known that large impacts can strongly affect the ecological environment by inducing tsunamis, fires and climate changes. The possibility of secondary catastrophes might affect the ecology via similar processes but with a lower magnitude of destruction because of the partial loss of the initial impact energy through the likely generation of strong earthquakes, tsunamis, and impact-induced magmatism.

### Short description of the used methods

For this study, field observations, Quadrocopter videographic and photographic observations (Mavic Pro, SZ DJI Technology Co., Ltd.) and microscopic structural and spectroscopic observations were obtained. The laboratory observations and measurements were performed via optical microscopy (POLAM-312, LOMO), scanning electron microscopy (SEM) (VEGA 3 TESCAN SEM with VEGA 3 LMN, INCA ENERGY 450 energy dispersive detector, Tescan, Czech Republic), and SEM with a focused ion beam (FIB/SEM) (Helios DualBeam, ThermoFisher Scientific, USA) at an accelerating voltage of 2 kV. The specimens for the TEM, STEM and EDX studies were prepared by a standard lift-out FIB technique. Microstructural analyses were performed in a Titan 80–300 TEM/STEM (ThermoFisher Scientific, USA) equipped with a spherical aberration corrector (probe corrector) with an accelerating voltage of 300 kV, and the STEM mode had a resolution of 0.08 nm. The device is equipped with an EDX Si(Li) spectrometer (EDAX, USA), a HAADF electron detector (Fischione, USA) and a Gatan Image Filter (GIF) (Gatan, USA). Image processing was performed using Digital Micrograph (Gatan, USA) and TIA (FEI, USA) software. Simulations of the EDX patterns and images were produced using Stadelmann’s EMS software package^58^.

## Supplementary information

Supplementary material.
