# Supplementary material for: Mysterious long-living ultrahigh-pressure or secondary impact crisis
Source: Sci Rep. 2020 Feb 13;10:2591. doi: 10.1038/s41598-020-59520-3 (PMC7018738; doi:10.1038/s41598-020-59520-3)
Supplement: Supplementary file 1 — Supplementary material. [file 41598_2020_59520_MOESM1_ESM.pdf]

Supplementary information

To the paper **"Mysterious long-living ultrahigh pressure or secondary impact crisis"**

T.G. Shumilova, A.A. Zubov, S.I.Isaenko, I.A.Karateev, A.L.Vasiliev

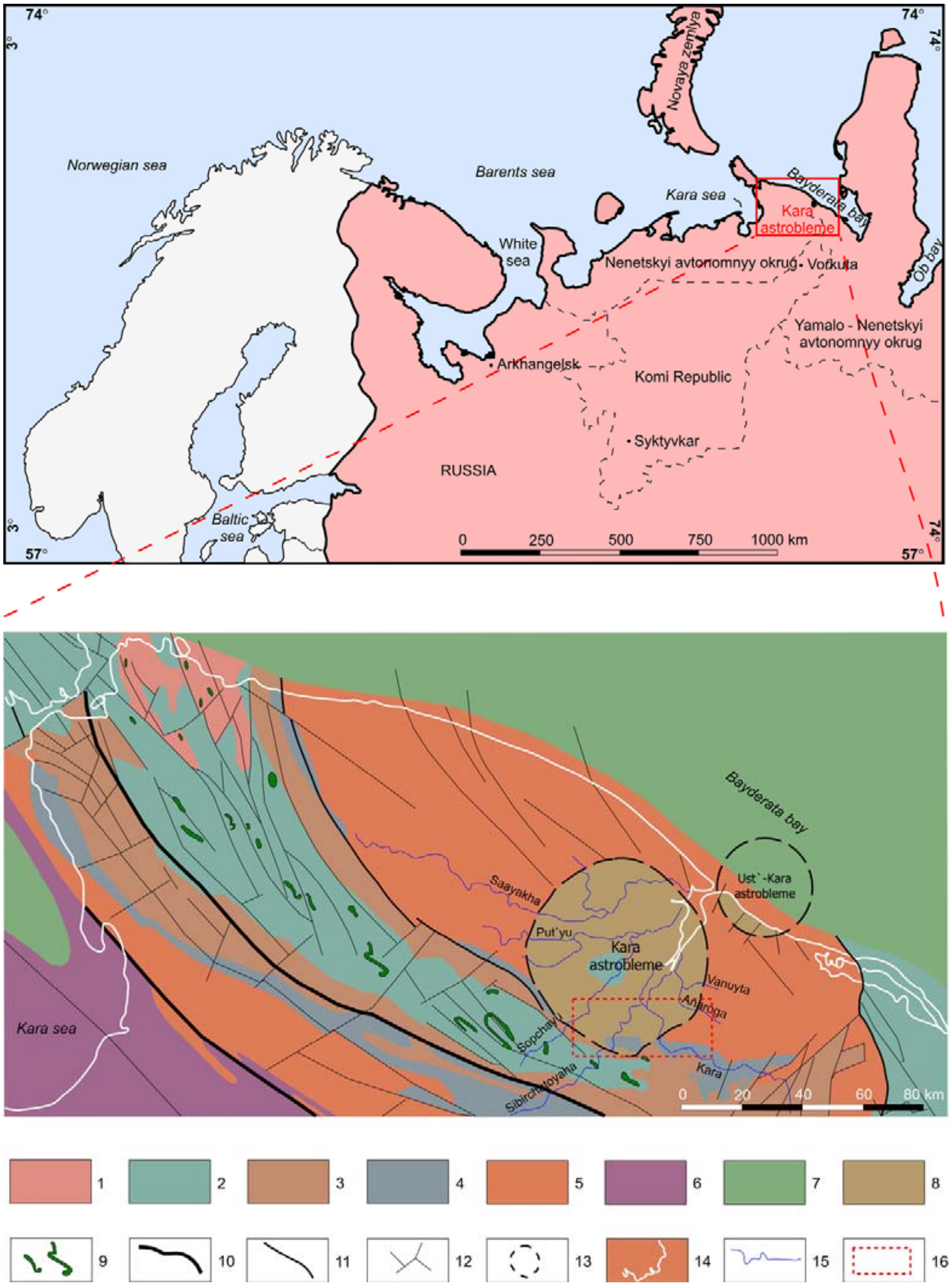

Figure 1. Geological scheme of the Kara region territory, simplified by S.I.Isaenko and T.G.Shumilova after the State Geological Map of Russia (GGK-1000) (reference: State Geological Map of Russia (GGK-1000), list R40(42), scale 1:1000000, Saint-Petersburg,

VSEGEI, 2000). Inset – geographic setting of the studied area. Sedimentary deposits: 1 – Upper Proterozoic; 2 – Silurian and Ordovician; 3 – Devonian, 4 – Carboniferous, 5 – Permian; 6 – Triassic; 7 – Cretaceous. Impactites – 8. Magmatic intrusions: 9 – Late Devonian tabular body and dikes of dolerite and gabbro-dolerite. Tectonic elements: 10 – deep faults; 11 – thrusts; 12 – small faults; 13 – boundary of astrobleme. Geographic elements: 14 – sea coast, 15 – rivers. 16 – sampling region at the Kara astrobleme. ***The figure is reprinted from the supporting material to the paper Shumilova T. G., Lutoev V. P., Isaenko S. I., Kovalchuk N. S., Makeev B. A., Lysiuk A. Yu., Zubov A. A. Spectroscopic features of ultrahigh-pressure impact glasses of the Kara astrobleme. Scientific Reports. V. 8. № 6923 (2018). DOI:10.1038/s41598-018-25037-z, https://www.nature.com/articles/s41598-018-25037-z with the permission of the Springer Nature.***

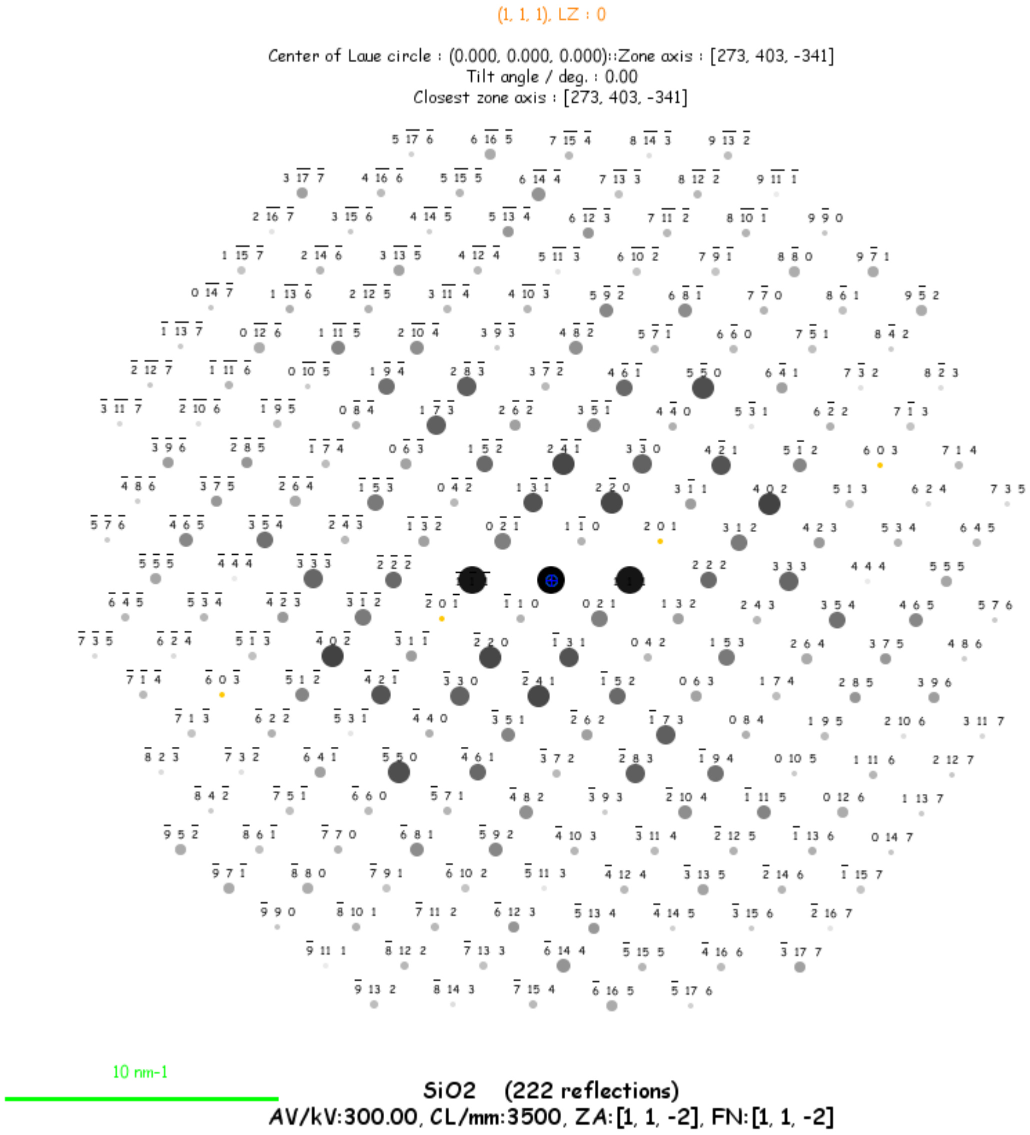

Figure 2. The simulated diffraction pattern to the figure 4 in the general text

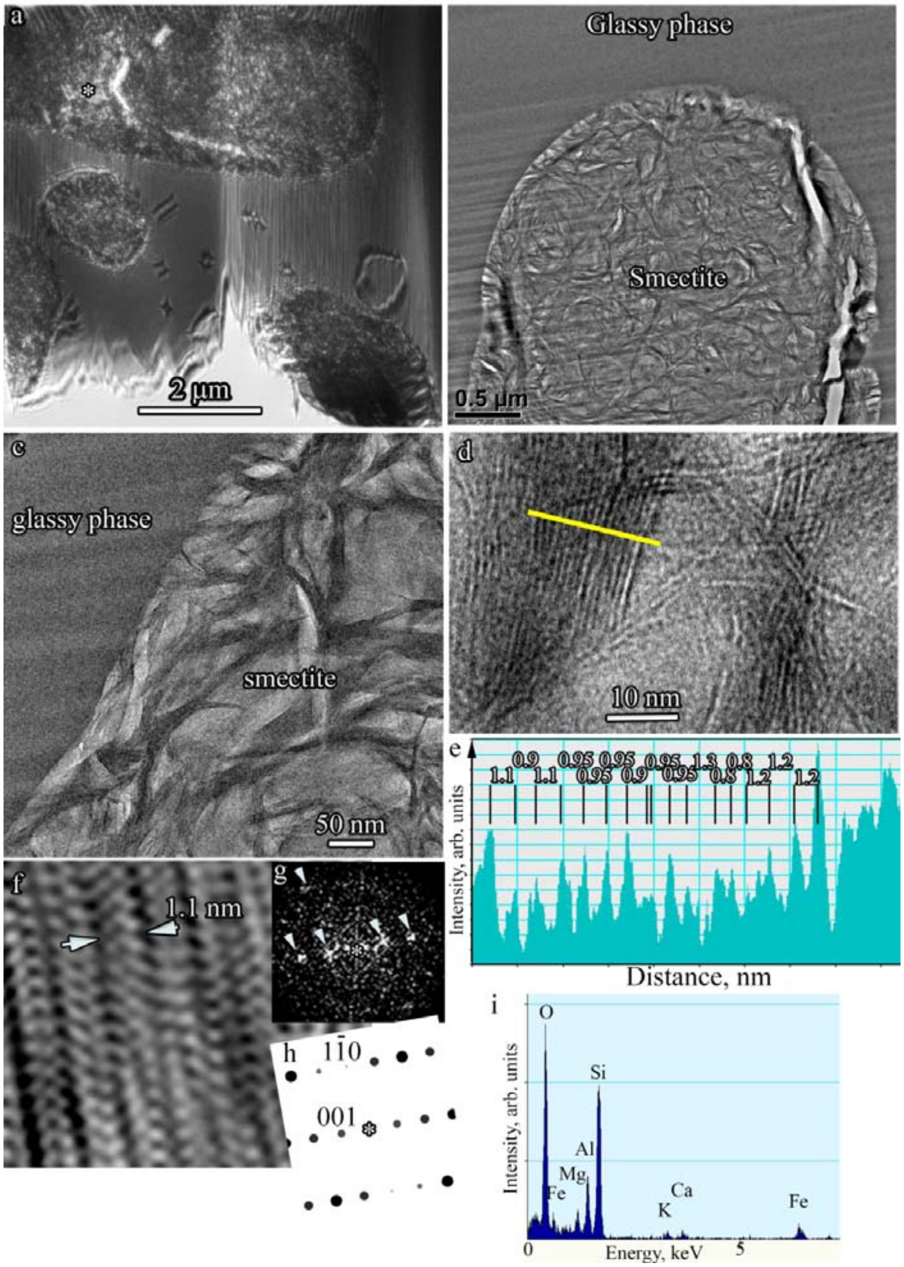

Figure 3. TEM data of smectite “drops” within the UHPHT glass: a – bright field (BF) overview image of the smectite “drops”; b – magnified BF image of a part of a smectite “drop”; c, d – higher magnified BF image of a part of a smectite “drop”, yellow line corresponds to HRTEM profile (e) of a smectite “drop”, f – HRTEM image of a smectite with insets of fast Furrier transformation (FFT) pattern and FFT pattern interpretation; i – X-Ray dispersive elemental analysis of smectite.

Table 1. Spot electron diffraction pattern data of a single crystalline coesite (to the fig. 4e)

Parameter	**Experimental data**	**Standard (table) data**
d(1-10)	0.5552 nm	0.5548 nm
d(021)	0.4389 nm	0.4376 nm
d(111)	0.3448 nm	0.3444 nm
angle((1-10),(021))	90.4°	90.3°
angle((1-10),(111))	52.0°	51.9°

Table 2. Microprobe data of associating phases within UHPHT vein glass, corresponding to the marked areas on the fig. 5e (atomic %)

**Area**	**O**	**Mg**	**Al**	**Si**	**Ca**	**Fe**
**Coesite**	**66.5**	**0.5**	**0.5**	**32.5**	**0**	**0**
**SiO_2 glass**	**66**	**0**	**0.5**	**33.5**	**0**	**0**
**Smectite**	**70**	**2**	**7**	**20**	**0.5**	**0.5**
